# Music reward sensitivity and subjective well-being in college students: the indirect pathways of resilience and perceived social support

**DOI:** 10.3389/fpsyg.2026.1897252

**Published:** 2026-08-13

**Authors:** Jinmei Tu, Qian Liu

**Affiliations:** School of Music and Dance, Guangzhou University, Guangzhou, Guangdong, China

**Keywords:** indirect pathway, music reward sensitivity, perceived social support, resilience, subjective well-being

## Abstract

**Background:**

Extensive research has shown that music is linked to subjective well-being. However, the specific role of music reward sensitivity seems to be neglected. The current study investigates the potential association between music reward sensitivity and the subjective well-being of college students, and further explores the indirect pathways of resilience and perceived social support.

**Methods:**

Using a multi-phase research design with two-month intervals, we surveyed 668 undergraduate participants across three waves, assessing music reward sensitivity, resilience, perceived social support, and the components of subjective well-being, including positive affect, negative affect, and life satisfaction. Hierarchical regression and indirect pathway analyses were used to examine the proposed associations.

**Results:**

Music reward sensitivity was positively associated with positive affect and life satisfaction, but was not significantly associated with negative affect. Indirect pathway analyses indicated that resilience and perceived social support were involved in the associations between music reward sensitivity and positive affect and life satisfaction. Moreover, the social reward facet of music reward sensitivity was positively associated with perceived social support, resilience, positive affect, and life satisfaction, but not with negative affect.

**Conclusion:**

This study highlights the value of considering music reward sensitivity in research on emotional well-being and life satisfaction among college students. The findings suggest that resilience and perceived social support may help explain the associations between music reward sensitivity and higher positive affect and life satisfaction. They also support future work testing whether music reward sensitivity, especially the socially rewarding aspects of music, can inform individualized music-related interventions.

## Background

Music is a potent source of happiness, universally woven into the fabric of human culture and daily life (Cook et al., 2019; Gustavson et al., 2021; Shan et al., 2024). Extensive studies have demonstrated the positive effects of music on emotional states and overall mood, showing that music not only evokes feelings of pleasure, happiness, and relaxation but also functions as a powerful tool to alleviate stress, reduce anxiety, and uplift one’s spirits (Haslam et al., 2009; Sekyung, 2021; Vigl et al., 2023; Wang and Suh, 2024; Zentner et al., 2008). Beyond emotion regulation, previous studies have found that music promotes social connection, strengthens cultural identity, and provides comfort during important life events (Fu and Tu, 2023; Pring et al., 2024; Piccolo et al., 2025). For example, shared musical experiences, such as participating in concerts, group singing, or communal rituals, can enhance collective happiness and a sense of belonging (Han et al., 2025; Yang et al., 2025). Across various age groups and cultures, music is consistently reported as a critical factor in supporting mental health and improving quality of life (Rentfrow et al., 2011). The diverse genres and styles of music enable people to choose what resonates most with their personal experiences and preferences, making music a highly individualized and accessible means of enhancing happiness (Rentfrow et al., 2011). Accordingly, understanding how music contributes to human happiness continues to be an important topic of research.

The capacity of music in evoking happiness in individuals stems from its intrinsic reward value, known as music reward. Music reward encapsulates the positive experiences and pleasure individuals derive when engaging with music (Salimpoor et al., 2011, 2013; Zatorre and Salimpoor, 2013). This concept involves multifaceted cognitive and emotional processes that aim to trigger emotional responses and evoke feelings of pleasure and positivity (Zatorre, 2015). Elements such as melody, harmony, rhythm, and lyrics play roles in this process, as they have the capacity to resonate emotionally with individuals, connecting with their personal experiences and inner thoughts (Snyder et al., 2024; Vuust et al., 2022). This emotional resonance facilitates the emergence of the experience of happiness and well-being in listeners. Neuroscience research on music has demonstrated the brain’s response to music stimuli, indicating that music can elicit specific patterns of activity in the reward network of the brain (Menon and Levitin, 2005). Specifically, when people are exposed to music that they find enjoyable or rewarding, regions within the brain’s reward network, including the nucleus accumbens, the ventromedial orbitofrontal cortex, and the striatum, become activated (Blood and Zatorre, 2001; Koelsch, 2014; Salimpoor et al., 2011, 2013; Zatorre and Salimpoor, 2013). These regions play a central role in releasing dopamine, a neurotransmitter closely linked to feelings of pleasure and reward (Berridge and Kringelbach, 2013; Pessiglione et al., 2006; Schultz, 2006). The release of dopamine in response to music engagement reinforces positive emotions, thereby contributing to the overall sense of well-being and happiness derived from musical experiences.

### Music reward sensitivity and subjective well-being

Existing literature has extensively focused on the positive effects of music on subjective well-being (SWB) (Dingle et al., 2021; Perkins et al., 2020; Tu and Fu, 2024). However, the role of music reward sensitivity appears to have been overlooked. Music reward sensitivity is the propensity to experience pleasure and positive emotions in response to musical stimuli, a trait that is underpinned by the neural reward system (Martínez-Molina et al., 2016; Mas-Herrero et al., 2013, 2018). Music reward sensitivity should be distinguished from general music engagement or listening frequency. Whereas music engagement describes how often or in what ways individuals participate in musical activities, music reward sensitivity refers to individual differences in the degree to which music is experienced as pleasurable, emotionally meaningful, and rewarding (Mas-Herrero et al., 2013; Martínez-Molina et al., 2016; Wang et al., 2023). Thus, two individuals may listen to music with similar frequency but differ substantially in the reward value they derive from music. This distinction is important because the present study focused on individual differences in music reward sensitivity rather than on the frequency or type of music engagement (Dingle et al., 2021; Gustavson et al., 2021). Individuals possess unique reward sensitivity to music, reflecting the diverse levels in which musical experiences impact their emotional well-being and overall satisfaction (Martínez-Molina et al., 2019; Mas-Herrero et al., 2014). These individual differences in music reward sensitivity are shaped by diverse factors, such as personal preferences and emotional connections to specific musical stimuli (Fuentes-Sánchez et al., 2022; Nieminen et al., 2011).

The association between music reward sensitivity and SWB can also be understood from the perspective of broaden-and-build theory. This theory suggests that positive emotional experiences can broaden individuals’ momentary thought-action repertoires and may support the development of adaptive psychosocial resources over time (Fredrickson, 1998, 2001, 2004; Fredrickson and Joiner, 2002). In the context of music, individuals with higher music reward sensitivity may be more likely to experience music as pleasurable, emotionally meaningful, and rewarding, which may be associated with higher positive affect and life satisfaction (Mas-Herrero et al., 2013; Salimpoor et al., 2013; Zatorre, 2015). This perspective is also consistent with research suggesting that positive emotions and positive affective experiences are related to psychological resources and subjective well-being (Diener et al., 2018). Therefore, broaden-and-build theory provides a useful background for understanding why music reward sensitivity may be related primarily to positive components of SWB, rather than necessarily to lower negative affect.

Sensitivity to specific music may be associated with enhancing liking and generating feelings of emotional comfort and contentment. Repeated exposure to familiar songs or musical pieces can evoke nostalgia and memories, thereby amplifying the perceived reward value of music (Herholz et al., 2012). Personal sensitivity and emotional connections to particular songs or artists may further enhance the contribution of music to one’s psychological well-being. Emotional links to important life events, meaningful relationships, or personal experiences imbue music with deep sentimental value and resonance (Jiang et al., 2023). As a result, individuals with emotional ties to specific music are more likely to derive profound levels of pleasure, comfort, and positive emotions from listening to these musical stimuli. In daily life, individuals with heightened music reward sensitivity are poised to derive greater subjective value and positive emotions from their musical experiences, exhibiting an increased responsiveness to the emotional cues and rewards embedded within music (Fuentes-Sánchez et al., 2021; Mas-Herrero et al., 2013). Such heightened sensitivity may enhance emotional experiences in the moment and support more effective emotion regulation. Consequently, those with greater music reward sensitivity may also exhibit higher SWB.

### The indirect roles of resilience and perceived social support

Resilience refers to the capacity to adapt positively and effectively to stress, adversity, or trauma (Southwick et al., 2014). It involves capabilities to bounce back from challenges, maintain feelings of well-being, and thrive under adversity. From a resilience theory perspective, adaptive functioning under stress is supported by personal resources, coping capacities, and social-contextual factors (Masten, 2011; Southwick et al., 2014). In this framework, resilience is not treated as a music-specific mechanism, but as a domain-general psychosocial resource that may be associated with rewarding musical experiences.

Music provides a platform for self-expression and emotional processing, all of which contribute to building personal resources that are essential for enhancing resilience (Perkins et al., 2020; Saarikallio et al., 2023). In particular, musical activities such as listening, performing, and songwriting can facilitate identity formation and the strengthening of self-efficacy during periods of adversity (Dingle et al., 2021; Han et al., 2025). Music is recognized as a powerful tool that can support and promote resilience in various ways. Specifically, music has the ability to regulate emotions, reduce stress, and provide comfort during difficult times (Peters et al., 2023; Saarikallio et al., 2023; Vigl et al., 2023). Engaging with music that resonates with individual preferences and emotions can be a coping mechanism, enabling people to process their feelings, find emotional release, and gain feelings of control over their emotional experiences (Cook et al., 2019; Gustavson et al., 2021; Vigl et al., 2023; Zentner et al., 2008). This emotional regulation through music can contribute to the improvement and maintenance of resilience, allowing people to cope more effectively with life’s difficulties (Nijs and Nicolaou, 2021; Rosenberg et al., 2021). Because rewarding musical experiences are often linked to emotion regulation and coping, individuals with higher music reward sensitivity may also report greater resilience, which may in turn be associated with more favorable well-being indicators.

Social support includes the wide array of assistance given by family, friends, neighbors, and others, involving a diverse range of social connections and interactions (Barrera et al., 1981). From a social support theory perspective, perceived support represents an important psychosocial resource that may be related to well-being by providing feelings of belonging, validation, and available assistance (Barrera et al., 1981; Cohen and Wills, 1985; Siedlecki et al., 2014). Perceived social support (PSS) is beneficial for individuals’ SWB, as it can buffer the negative effects and improve life satisfaction (Siedlecki et al., 2014). In the present study, PSS was therefore examined as a domain-general social resource that may be involved in the association between music reward sensitivity and positive well-being indicators.

Music has the ability to evoke emotions, create connections, and foster a sense of belonging, all of which are essential components of social support (Koelsch, 2014). Engaging with music that resonates with personal preferences can be a source of emotional comfort and connection, helping individuals feel understood, validated, and supported (Yang et al., 2025). Through engagement with music, individuals may perceive the experience of support from artists, lyrics, or fellow listeners, creating a virtual network of emotional connectivity and understanding (Rabinowitch, 2020). This perceived support can enhance individuals’ overall well-being by providing them with the experience of security, validation, and belonging (Dingle et al., 2021). In this way, music can simulate the experience of social support by providing a platform for self-expression, empathy, and shared experiences (Perkins et al., 2020; Piccolo et al., 2025). By encouraging social interaction and enhancing social ties, music contributes directly to the core aspects of PSS (D’Ausilio et al., 2015; Savage et al., 2021). Participating in musical activities, such as singing in choirs, attending concerts, or engaging in music therapy sessions, can promote feelings of community, camaraderie, and shared experience among individuals (D’Ausilio et al., 2015). These shared musical experiences create opportunities for bonding, empathy, and collective support, enhancing individuals’ sense of social connectedness and perceived support. Consequently, PSS may represent one pathway linking music reward sensitivity with positive well-being indicators.

This proposed social pathway is also consistent with the view that music reward sensitivity is multidimensional (Mas-Herrero et al., 2013; Wang et al., 2023). Beyond individual pleasure, nostalgia, and emotion regulation, music can be rewarding because it is socially meaningful and connected to interpersonal experiences (Dingle et al., 2021). Socially rewarding musical experiences may be associated with feeling understood, connected, and supported, especially when music is shared with others or linked to important relationships and group experiences (Han et al., 2025; Yang et al., 2025). Recent work on communal music similarly suggests that shared or socially meaningful music experiences are associated with belonging, social connectedness, and positive affect (Piccolo et al., 2025). Therefore, the social component of music reward sensitivity may be particularly relevant to the present model linking music reward sensitivity with perceived social support and positive well-being indicators.

### The current study

Taken together, the proposed model was informed by the music reward framework, broaden-and-build theory, resilience theory, and social support theory. The music reward framework suggests that individuals differ in the extent to which they experience music as pleasurable, emotionally meaningful, and rewarding (Mas-Herrero et al., 2013; Martínez-Molina et al., 2016; Zatorre, 2015). Broaden-and-build theory further suggests that positive emotional experiences may broaden individuals’ thought-action repertoires and be linked to adaptive psychosocial resources over time (Fredrickson, 1998, 2001, 2004; Fredrickson and Joiner, 2002). In the present context, individuals with higher music reward sensitivity may be more likely to report positive well-being indicators because rewarding musical experiences are closely related to pleasure, affective reward, and emotion regulation. From a resilience theory perspective, coping-related personal resources are central to adaptation under stress and adversity (Masten, 2011; Southwick et al., 2014). From a social support theory perspective, perceived support represents an important psychosocial resource related to well-being and stress adaptation (Barrera et al., 1981; Cohen and Wills, 1985; Siedlecki et al., 2014). Accordingly, resilience and PSS were examined as domain-general psychosocial resources that may be involved in the associations between music reward sensitivity and SWB components.

Although previous research has extensively investigated associations between music and SWB among college students, less attention has been paid to music reward sensitivity as an individual-difference factor and to the potential indirect pathways involving resilience and PSS. Therefore, this study aimed to examine whether music reward sensitivity was associated with the components of SWB among college students and whether resilience and PSS were involved in these associations. Because SWB was assessed through positive affect, negative affect, and satisfaction with life, these three components were treated as the primary outcomes.

**Figure1:**
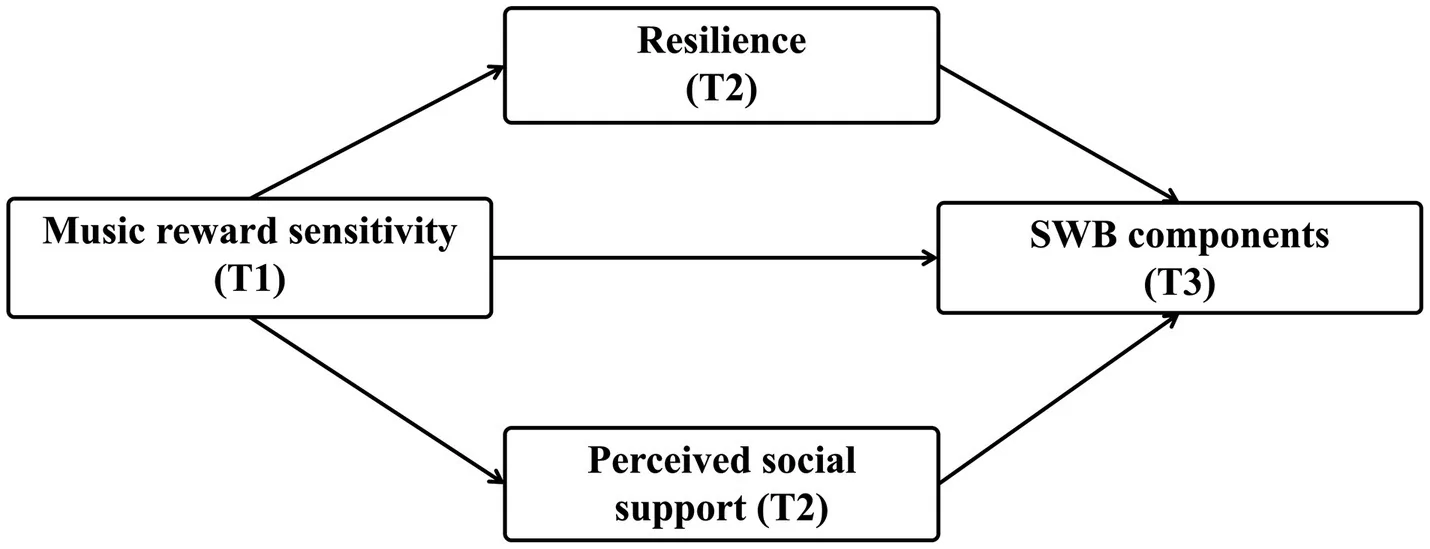
Conceptual model of the temporally ordered associative pathways.

The conceptual model is presented in Figure 1. The model was intended to represent temporally ordered associative pathways rather than causal mediation.

*H1*: Music reward sensitivity will be associated with the components of SWB.

*H2*: Resilience will be involved in the indirect pathways linking music reward sensitivity with SWB indicators.

*H3*: Perceived social support will be involved in the indirect pathways linking music reward sensitivity with SWB indicators.

In addition, we conducted supplementary exploratory facet-level analyses of music reward sensitivity, with particular attention to the social reward facet.

## Method

### Study design and participants

This study used an online questionnaire website^1^ to collect the data at three different time points, with a two-month interval between each data collection period. During the initial phase of data collection (T1), we measured participants’ music reward sensitivity and demographic information. In the subsequent stage of data collection (T2), participants’ resilience and PSS were obtained. At the T3, participants completed measures of the components of SWB, including positive affect (PA), negative affect (NA), and satisfaction with life (SWL).

Prior to each data collection, all participants provided informed consent and received explicit information regarding the voluntary nature of the study and their right to withdraw at any time. Additionally, participants were told that their personal data would remain confidential and be protected throughout the study. None of the participants reported any psychiatric disorders or a history of psychiatric disorders. All participants received compensation after each data collection. We conducted an *a priori* power analysis using the G*Power software (Faul et al., 2007) to estimate the required sample size, with a medium effect size *f* ^2^ = 0.15 and significant level *α* = 0.05. A minimum sample size of 146 participants was determined to be necessary to provide 95% statistical power (i.e., 1-*β*) for this study.

Participants were recruited through convenience sampling from undergraduate students at several universities in Guangzhou, including Guangzhou University, South China Normal University, Sun Yat-sen University, and South China University of Technology. We initially recruited 707 college students to participate in the data collection at T1. Among these participants, a subset of 674 students maintained their engagement in the survey at T2. Upon concluding the data collection at T3, it was determined that only 668 students successfully completed all three questionnaires. Participants were between 18 and 24 years old (*M*_age_ = 19.87 ± *SD* = 0.90), with 61.4% being female and 38.6% male. Regarding their grade levels, 8 participants (1.2%) were classified as freshmen, 250 participants (37.4%) as sophomores, 401 participants (60.0%) as juniors, and 9 participants (1.3%) as seniors. In terms of their fields of study, 370 participants (55.4%) were pursuing degrees in natural science and engineering, 290 participants (43.4%) in humanities and social sciences, and 8 participants (1.2%) in art and aesthetics. Furthermore, 154 participants (23.1%) reported being only children, while 514 participants (76.9%) indicated having siblings.

### Measurements

All instruments used in the present study were Chinese versions that had been previously translated, adapted, or psychometrically validated in Chinese samples. In the current sample, internal consistency reliability was evaluated using Cronbach’s alpha.

#### Barcelona music reward questionnaire

Music reward sensitivity in this research was assessed with the Barcelona Music Reward Questionnaire (BMRQ) (Martínez-Molina et al., 2016). The Chinese adaptation of the BMRQ, translated and validated by Wang et al. (2023), includes 16 items, with an example item being “I like to listen to music that contains emotion.” Participants responded to each statement using a five-point Likert scale from 1 (strongly disagree) to 5 (strongly agree). Higher scores indicate greater subjective reward and positive emotional responses derived from engaging with and appreciating music in daily life. The Cronbach’s alpha coefficient for the BMRQ was 0.89.

For supplementary facet-level analyses, the 16 BMRQ items were scored according to the five dimensions of the Chinese BMRQ: musical seeking, emotional evocation, mood regulation, sensory-motor, and social reward (Mas-Herrero et al., 2013; Wang et al., 2023). In the present sample, the facet-level descriptive statistics and reliability estimates were as follows: musical seeking (*M* = 7.51, *SD* = 1.42, Cronbach’s alpha = 0.47), emotional evocation (*M* = 16.27, *SD* = 2.33, Cronbach’s alpha = 0.69), mood regulation (*M* = 16.71, *SD* = 2.13, Cronbach’s alpha = 0.79), sensory-motor (*M* = 8.45, *SD* = 1.34, Cronbach’s alpha = 0.73), and social reward (*M* = 15.00, *SD* = 2.46, Cronbach’s alpha = 0.71). The social reward facet was emphasized because of its theoretical relevance to perceived social support. Because musical seeking consisted of only two items and showed relatively low internal consistency, facet-level findings should be interpreted cautiously.

#### Multidimensional scale of perceived social support

PSS was assessed using the Multidimensional Scale of Perceived Social Support (MSPSS; Zimet et al., 1988). Chen et al. (2017) adapted and validated a Chinese version of the MSPSS for school students, which contains 12 items (e.g., “I can talk about my problems with my friends”). Each item is rated on a 7-point Likert scale ranging from 1 (“strongly disagree”) to 7 (“strongly agree”). Higher scores indicate greater perceived social support from others in daily life. The MSPSS demonstrated excellent reliability, with a Cronbach’s alpha of 0.947.

#### Connor–Davidson resilience scale

Resilience was measured using the Connor-Davidson Resilience Scale (CD-RISC), consisting of 10 items as a brief version of the original version with 25 items (Campbell-Sills and Stein, 2007; Connor and Davidson, 2003). The Chinese adaptation of CD-RISC was translated and psychometrically verified by Wang et al. (2010). The CD-RISC demonstrates good measurement properties with a single-factor structure. A representative item from the scale is “I think of myself as a strong person.” Each item in the CD-RISC was rated by participants on a scale ranging from 1 (“not true at all”) to 5 (“true nearly all the time”). Higher scores indicate individuals’ greater capacity to adapt and cope with stress in their daily lives. The CD-RISC exhibited excellent reliability, with a Cronbach’s alpha of 0.923.

#### Positive and negative affect schedule

The PA and NA were evaluated using the Positive and Negative Affect Schedule (PANAS) (Watson et al., 1988). The Chinese adaptation of PANAS was revised and verified by Qiu et al. (2008). The scale consisted of nine items assessing positive emotional experiences (e.g., “excited”) and nine items assessing negative emotional experiences (e.g., “distressed”). Participants rated how often they experienced these emotions in their daily lives on a 5-point Likert scale, from 1 (“not at all”) to 5 (“extremely”). Higher PA scores indicate more frequent positive affective experiences, whereas higher NA scores indicate more frequent negative affective experiences. The Cronbach’s alpha coefficients for the PA and NA subscales were 0.934 and 0.896, respectively.

#### Satisfaction with life scale

The SWL was measured using the Satisfaction with Life Scale (SWLS) (Diener et al., 1985). Xiong and Xu (2009) have translated and verified the Chinese version of SWLS. The scale consisted of five items with a single-factor, such as “I am satisfied with my life.” Participants rated all the items on a 7-point Likert scale, ranging from 1 (strongly disagree) to 7 (strongly agree). Higher scores indicate a greater level of SWL. The Cronbach’s alpha coefficient for the SWLS was 0.859.

#### Control variables

We included five demographic variables (i.e., gender, age, grade, only-child status, and major) as control variables for analysis. Age was treated as a continuous variable. Gender and only-child status were both coded as dummy variables (gender: male = 0, female = 1; only-child status: only child = 0, non-only child = 1). Grade was categorized into four levels as “1” for freshman (first grade), “2” for sophomore (second grade), “3” for junior (third year), and “4” for senior (fourth grade). Similarly, major was coded as a categorical variable, with “1” representing natural science and engineering majors, “2” representing humanities and social science majors, and “3” representing art and aesthetics majors.

### Data analysis

The primary independent variable was overall music reward sensitivity, while resilience and PSS were examined as indirect pathway variables. The primary dependent variables were the three components of SWB: positive affect, negative affect, and satisfaction with life. Hierarchical regression analyses were conducted to examine associations between music reward sensitivity and the primary outcomes after controlling for demographic variables. In the first step of regression models, control variables (i.e., gender, age, grade, only-child status, and major) were entered. In the second step, the independent variable was added, which enabled the assessment of its unique association with the dependent variables. Indirect pathway analyses were conducted using the PROCESS macro (version 3.4.1, Model 4) to estimate parallel indirect pathways (Hayes, 2022). Control variables were included as covariates in all indirect pathway analyses. Consistent with Hayes’ suggestion (Hayes, 2022), PROCESS analyses were conducted using 5,000 iterations (i.e., bootstrap sampling) and 95% confidence intervals (CI). Because each construct was measured once, these models were interpreted as temporally ordered associative pathway models rather than evidence of causal mediation or within-person change.

To examine the theoretically relevant social component of music reward sensitivity, we conducted supplementary analyses focusing on the social reward facet of the BMRQ. Hierarchical regression models with demographic covariates were used to examine whether social reward was associated with resilience, PSS, PA, NA, and SWL. To test whether these associations were specific to social reward rather than reflecting shared variance with other BMRQ facets, all five BMRQ facets were entered simultaneously, with social reward as the focal facet. In addition, exploratory indirect pathway models were estimated using social reward as the focal independent variable.

## Results

### Descriptive and correlation analyses

Table 1 presents the descriptive statistics (i.e., means and standard deviations) and correlations for all study variables. Consistent with expectations, music reward sensitivity showed significant positive associations with PSS, resilience, PA, and SWL (all |*r|*s ≥ 0.20, all *p*s < 0.001). In contrast, no significant association was found between music reward sensitivity and NA (*r* = −0.03, *p* = 0.374). Notably, both resilience and PSS were significantly related to PA, NA, and SWL (all |*r|*s ≥ 0.18, all *p*s < 0.001). These patterns of association provided a basis for further hypothesis testing.

**Table 1:** Descriptive statistics and bivariate correlations (*N* = 668).

Variable	*M* ± *SD*	1	2	3	4	5	6	7	8	9	10
1. Age	19.88 ± 0.96	–									
2. Gender	0.61 ± 0.49	−0.11**	–								
3. Only-child status	0.77 ± 0.42	−0.04	0.14***	–							
4. Major	1.46 ± 0.52	−0.01	0.37***	0.04	–						
5. Grade	2.62 ± 0.54	0.48***	−0.10**	−0.04	0.01	–					
6. Music reward	63.96 ± 7.90	−0.06	0.07	−0.07	0.16***	−0.08**	–				
7. Resilience	35.06 ± 6.16	−0.04	−0.13**	−0.03	−0.05	−0.05	0.32***	–			
8. Perceived social support	61.06 ± 11.22	−0.05	0.04	−0.05	0.08*	−0.01	0.33***	0.53***	–		
9. Positive affect	30.05 ± 6.54	−0.07	0.01	0.000	0.03	−0.06	0.30***	0.36***	0.38***	–	
10. Negative affect	21.61 ± 6.37	−0.04	−0.01	0.09*	0.08*	−0.002	−0.03	−0.24***	−0.18***	−0.20***	–
11. Satisfaction with life	21.72 ± 5.36	−0.06	−0.01	−0.12**	0.04	−0.001	0.21***	0.42***	0.46***	0.57***	−0.32***

*p < 0.05, **p < 0.01, ***p < 0.001.

### Hierarchical regression analyses

The hierarchical regression analyses are presented in Table 2. The results showed positive associations of music reward sensitivity with PA and SWL, even after controlling for demographic variables (both *β*s ≥ 0.20, both *p*s < 0.001). These results provide partial evidence for H1. Nevertheless, music reward sensitivity was not significantly associated with NA when controlling for demographic variables (*β* = −0.04, *p* = 0.281). Therefore, subsequent indirect pathway analyses focused on PA and SWL.

**Table 2:** Hierarchical regression analyses examining associations with SWB components.

Dependent variable	Step	Predictor	*b*	SE	*β*	*t*	*p*
Positive affect	Model 1	Age	−0.35	0.30	−0.05	−1.17	0.244
	*R*^2^ = 0.01	Gender	−0.13	0.57	−0.01	−0.23	0.819
		Only-child status	−0.05	0.61	0.00	−0.09	0.931
		Major	0.41	0.52	0.03	0.79	0.432
		Grade	−0.42	0.54	−0.03	−0.77	0.440
	Model 2	Age	−0.30	0.29	−0.04	−1.05	0.296
	Δ *R*^2^ = 0.09	Gender	−0.21	0.55	−0.02	−0.39	0.698
		Only-child status	0.32	0.58	0.02	0.56	0.579
		Major	−0.15	0.51	−0.01	−0.30	0.767
		Grade	−0.16	0.52	−0.01	−0.31	0.760
		Music reward	0.25	0.03	0.30	7.88	<0.001
Negative affect	Model 1	Age	−0.34	0.29	−0.05	−1.14	0.253
	*R*^2^ = 0.02	Gender	−0.77	0.55	−0.06	−1.39	0.166
		Only-child status	1.35	0.59	0.09	2.29	0.022
		Major	1.22	0.51	0.10	2.40	0.017
		Grade	0.23	0.52	0.02	0.43	0.664
	Model 2	Age	−0.34	0.29	−0.05	−1.17	0.244
	Δ *R*^2^ = 0.002	Gender	−0.75	0.55	−0.06	−1.37	0.173
		Only-child status	1.29	0.59	0.09	2.19	0.029
		Major	1.29	0.51	0.11	2.52	0.012
		Grade	0.19	0.52	0.02	0.37	0.715
		Music reward	−0.03	0.03	−0.04	−1.08	0.281
Satisfaction with life	Model 1	Age	−0.43	0.25	−0.08	−1.74	0.083
	*R*^2^ = 0.02	Gender	−0.24	0.46	−0.02	−0.51	0.608
		Only-child status	−1.48	0.49	−0.12	−3.00	0.003
		Major	0.55	0.43	0.05	1.29	0.198
		Grade	0.28	0.44	0.03	0.65	0.519
	Model 2	Age	−0.40	0.24	−0.07	−1.66	0.098
	Δ *R*^2^ = 0.04	Gender	−0.28	0.46	−0.03	−0.62	0.536
		Only-child status	−1.28	0.49	−0.10	−2.63	0.009
		Major	0.25	0.42	0.02	0.58	0.563
		Grade	0.42	0.43	0.04	0.98	0.327
		Music reward	0.13	0.03	0.20	5.10	<0.001

Unstandardized coefficients are presented as b. Standardized coefficients are presented as β. Standard errors are presented as SE.

Supplementary regressions focusing on social reward showed a parallel pattern. After controlling for age, gender, only-child status, major, and grade, social reward was significantly associated with resilience (*β* = 0.353, *p* < 0.001), PSS (*β* = 0.327, *t* = 8.75, *p* < 0.001), PA (*β* = 0.303, *p* < 0.001), and SWL (*β* = 0.241, *p* < 0.001). Its association with NA did not reach significance (*β =* −0.072, *p* = 0.069). When all five BMRQ facets were entered simultaneously, social reward remained significantly associated with resilience (*β* = 0.279, *p* < 0.001), PSS (*β* = 0.249, *p* < 0.001), PA (*β* = 0.226, *p* < 0.001), and SWL (*β* = 0.237, *p* < 0.001). These findings suggest that the social component of music reward was not merely redundant with the other BMRQ facets.

### Indirect pathway analyses

**Figure 2:**
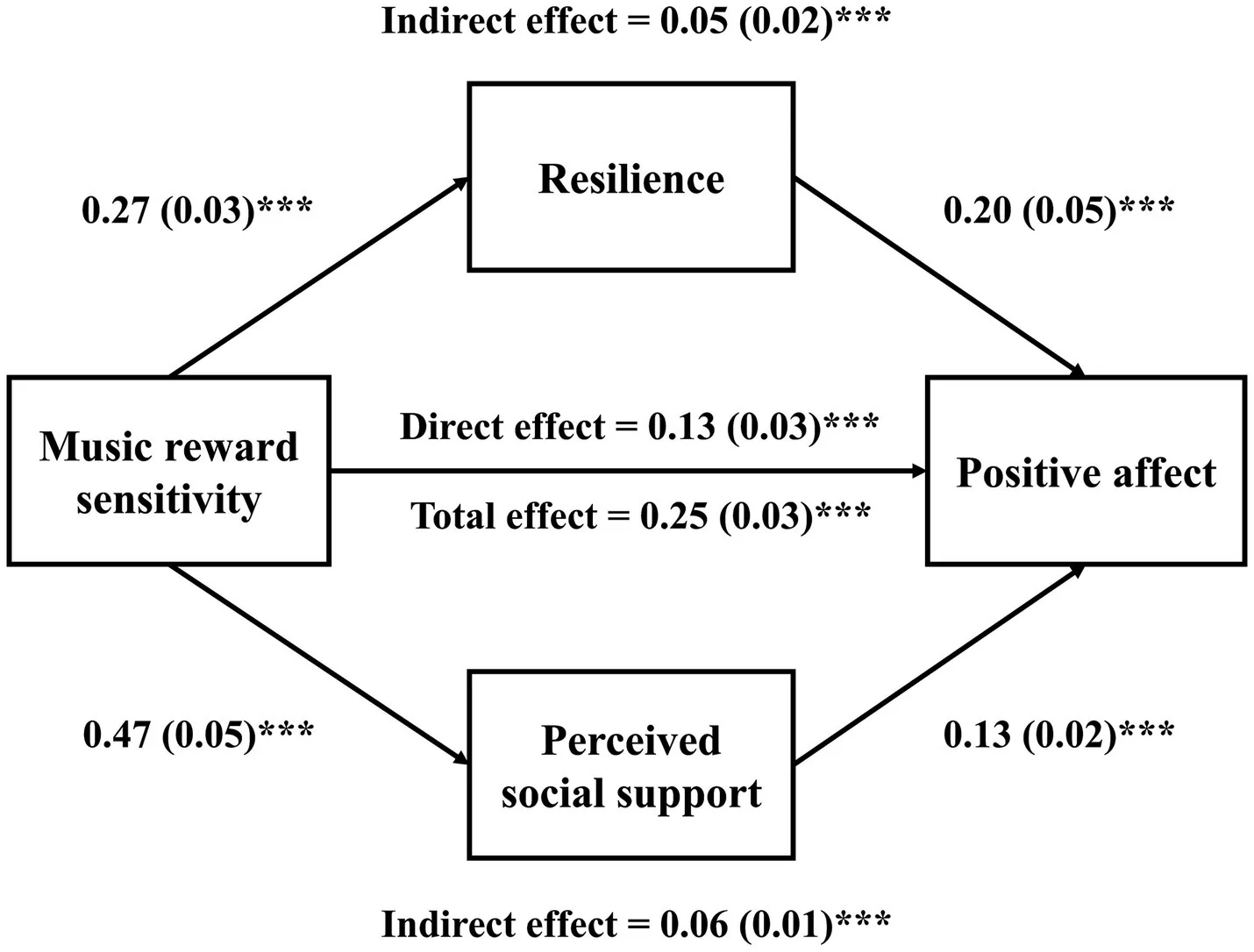
Indirect pathway model linking music reward sensitivity with PA via resilience and PSS. ***p < 0.001.

Indirect pathway models were conducted to examine whether resilience and PSS were involved in the associations between music reward sensitivity and the primary SWB outcomes. Because music reward sensitivity was not significantly associated with NA, indirect pathway analyses were conducted for PA and SWL. The indirect pathway model with PA as the dependent variable is shown in Figure 2. The total effect of music reward sensitivity on PA was significant (*b* = 0.25, *t* = 7.88, *p* < 0.001). The direct effect of music reward sensitivity on PA was also significant (*b* = 0.13, *t* = 4.19, *p* < 0.001). Music reward sensitivity was positively associated with resilience (*b* = 0.27, *t* = 9.19, *p* < 0.001) and PSS (*b* = 0.46, *t* = 8.60, *p* < 0.001). Both resilience (*b* = 0.20, *t* = 4.47, *p* < 0.001) and PSS (*b* = 0.13, *t* = 5.41, *p* < 0.001) were positively associated with PA. Moreover, the indirect effects of resilience (indirect effect = 0.05, 95% CI = [0.02, 0.09]) and PSS (indirect effect = 0.06, 95% CI = [0.03, 0.09]) on the relationship between music reward sensitivity and PA were both significant. These results provide partial evidence for H2 and H3.

**Figure 3:**
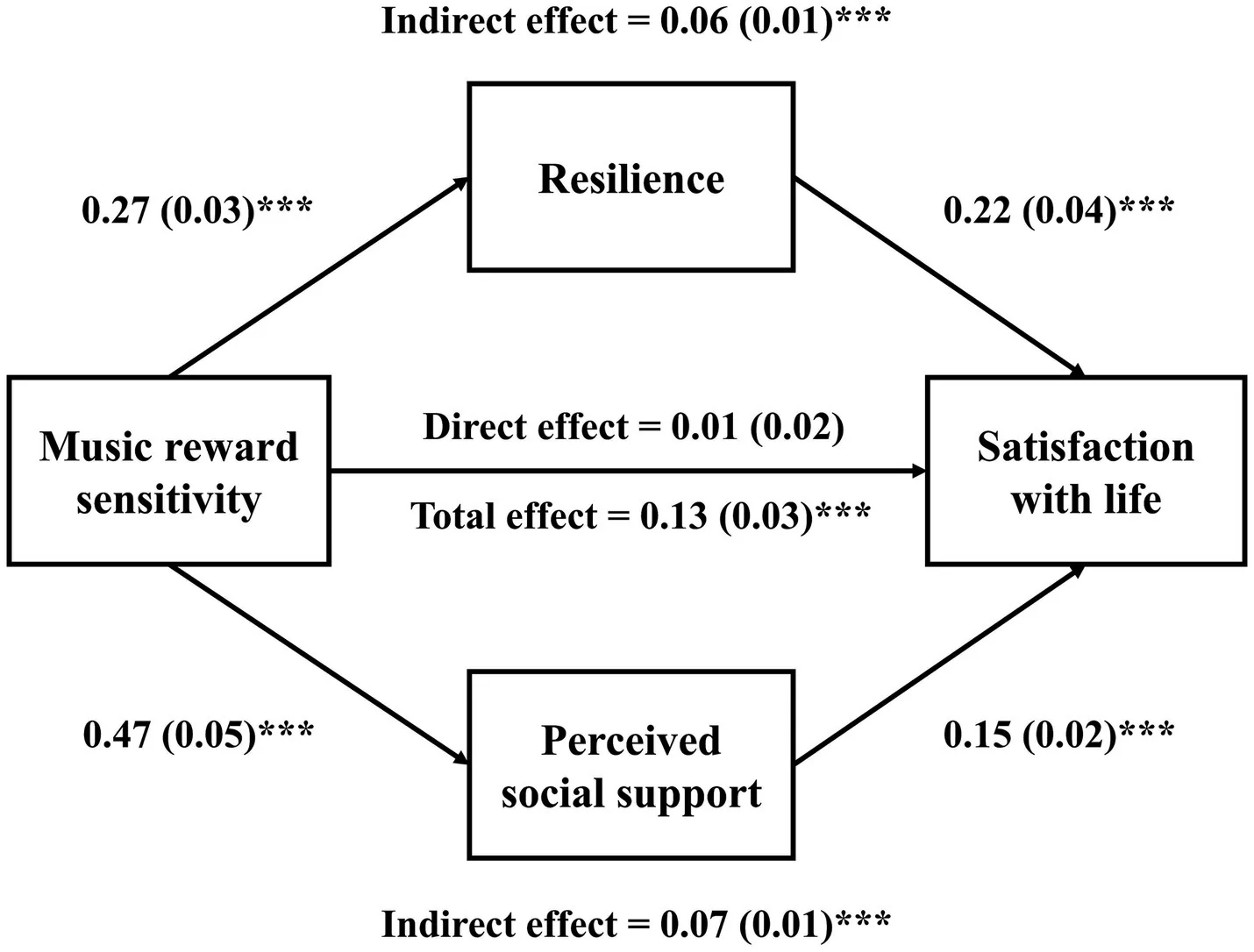
Indirect pathway model linking music reward sensitivity with SWL via resilience and PSS. ***p < 0.001.

The indirect pathway model with SWL as the outcome variable is shown in Figure 3. The total effect of music reward sensitivity on SWL was significant (*b* = 0.13, *t* = 5.10, *p* < 0.001). However, the direct effect of music reward sensitivity on SWL was not significant (*b* = 0.01, *t* = 0.31, *p* = 0.757). Music reward sensitivity was positively associated with resilience (*b* = 0.27, *t* = 9.19, *p* < 0.001) and PSS (*b* = 0.46, *t* = 8.60, *p* < 0.001). Both resilience (*b* = 0.22, *t* = 6.13, *p* < 0.001) and PSS (*b* = 0.15, *t* = 7.65, *p* < 0.001) were positively associated with SWL. Moreover, the indirect effects of resilience (indirect effect = 0.06, 95% CI = [0.03, 0.09]) and PSS (indirect effect = 0.07, 95% CI = [0.04, 0.09]) on the relationship between music reward sensitivity and SWL were both significant. These results provide partial evidence for H2 and H3.

Exploratory indirect pathway models using social reward as the focal independent variable showed a similar pattern. For PA, the indirect effects through resilience (indirect effect = 0.176, 95% CI = [0.078, 0.292]) and PSS (indirect effect = 0.198, 95% CI = [0.110, 0.293]) were both significant. For SWL, the indirect effects through resilience (indirect effect = 0.185, 95% CI = [0.110, 0.272]) and PSS (indirect effect = 0.215, 95% CI = [0.142, 0.295]) were also significant.

## Discussion

Using data from 668 participants across three waves, this study examined whether music reward sensitivity was associated with the components of SWB among college students and whether resilience and PSS were involved in these associations. Consistent with our hypotheses and previous research, the results showed that music reward sensitivity was positively associated with PA and SWL. In addition, resilience and PSS statistically accounted for part of these associations, suggesting that heightened music reward sensitivity is related to higher resilience and perceived social support, which are also related to higher positive affect and life satisfaction. Notably, the association between music reward sensitivity and NA was not significant, indicating that music reward sensitivity may be more closely related to positive dimensions of well-being than to lower negative affect.

Our results are consistent with previous research on the positive relationship between music and SWB (Gustavson et al., 2021; Shan et al., 2024; Tu and Fu, 2024; Mas-Herrero et al., 2013), and extend prior work by demonstrating the significance of individual sensitivity to music-induced rewards. Importantly, this study highlights that individuals differ in the extent to which they report positive emotional and psychological correlates of musical reward. Students with higher music reward sensitivity tended to report higher positive emotional and psychological well-being indicators. Theoretically, music reward sensitivity represents an individual’s propensity to derive pleasure and affective reward from musical experiences, which is rooted in the neurobiology of the brain’s reward system (Martínez-Molina et al., 2016; Mas-Herrero et al., 2013, 2018). When music stimulates the brain’s dopaminergic reward circuits, particularly in highly sensitive individuals, it may lead to more substantial experiences of joy and meaning (Salimpoor et al., 2013). This reward-related perspective provides a basis for understanding why music reward sensitivity may be associated with positive well-being indicators (Menon and Levitin, 2005; Zatorre, 2015).

These findings further clarify the indirect pathway involving resilience. Students with higher music reward sensitivity reported higher resilience, and higher resilience was related to higher PA and SWL. Previous research suggests that music can contribute to emotional regulation and stress coping, which are key components of resilience (Hakim et al., 2023; Sekyung, 2021; Wang and Suh, 2024). This pattern is consistent with prior work suggesting that rewarding musical experiences are related to positive affect, emotion regulation, and coping-related resources (Menon and Levitin, 2005; Rosenberg et al., 2021; Salimpoor et al., 2013; Zatorre, 2015). However, because resilience was measured only once and was assessed as a domain-general construct, these findings should be interpreted as evidence of a temporally ordered indirect pathway rather than as evidence that music reward sensitivity increases resilience.

In addition, our results indicate that PSS may represent another indirect pathway in the relationship between music reward sensitivity and positive components of SWB. Previous studies have shown that shared musical experiences, such as participating in group music-making activities or attending concerts, can create opportunities for social connection and bonding (Perkins et al., 2020; Siedlecki et al., 2014). These communal experiences may be associated with greater feelings of belonging and support (Dingle et al., 2021; Rabinowitch, 2020). When individuals perceive music as a meaningful source of support and connection, they may be more likely to report feelings of belonging, validation, and comfort (Han et al., 2025). Higher music reward sensitivity was associated with greater PSS, which in turn was related to more favorable positive well-being indicators. The social reward findings further clarify this interpretation. Among the BMRQ facets, social reward was directly aligned with perceived social support and remained significant when the other music reward facets were included in the same model. This pattern suggests that the social meaning of musical reward may be particularly relevant to the PSS pathway, beyond music reward sensitivity as a global tendency. Together, these findings suggest that perceived social support and the social meaning of musical reward are relevant to positive well-being indicators among college students.

Interestingly, this study revealed no significant relationship between music reward sensitivity and NA, despite the positive associations observed with resilience, PSS, SWL, and PA. This finding may suggest that while music reward sensitivity appears to be connected mainly with positive emotional states and satisfaction, it does not necessarily correspond to lower levels of negative emotions. One possible explanation is that positive and negative emotions are, to some extent, governed by different psychological mechanisms, reflecting the independence and distinctiveness of SWB constructs (Diener et al., 2018). Music reward sensitivity may be more relevant for amplifying positive emotions rather than attenuating negative states. Thus, the relationship between music, affect, and well-being is complex, potentially moderated by the type and context of musical engagement, as well as individual differences. Future research should further examine these potential contributing factors to better understand how music reward sensitivity relates to different components of well-being.

Several limitations of this study should be noted. First, although the three-wave design provided temporal separation among variables, each construct was measured only once, and baseline levels of resilience, PSS, PA, NA, and SWL were not controlled. Therefore, the indirect pathway models should not be interpreted as evidence of causal mediation or within-person change. Second, the sample comprised only Chinese college students, which raises concerns about the generalizability of the findings to other age groups, cultural contexts, and clinical populations with varied musical backgrounds or stress levels. Cross-cultural studies are therefore warranted to determine whether these findings hold more broadly. Third, while this study focused on music reward sensitivity as a personality trait, other individual differences, such as personality dimensions, musical background, or different forms of musical engagement, including active music-making versus passive listening, were not examined. Future research could further explore potential moderators (e.g., genre preference, use of music for emotion regulation, baseline emotional health) using more comprehensive measures or mixed-method approaches. Fourth, the facet-level analyses should be interpreted as supplementary and exploratory. Although they helped clarify the theoretical relevance of social reward, they were not part of the original primary hypotheses. Future studies should preregister facet-level hypotheses and include music-specific measures of belonging, shared musical activity, and social reward. Finally, much remains unknown regarding the neural foundations of these associations. Neuroimaging and psychophysiological studies could help clarify how music reward processes interact with resilience and social support networks in the brain.

Overall, this study highlights the value of considering music reward sensitivity in research on emotional well-being and life satisfaction among college students. Higher music reward sensitivity was associated with higher PA and SWL, and these associations were linked to indirect pathways involving resilience and PSS. The findings also suggest that the social reward facet of music reward sensitivity may be especially relevant to perceived social support and positive well-being indicators. Future experimental and longitudinal studies are needed to test whether music reward sensitivity, especially social reward from music, can inform individualized music-related interventions.

## Data Availability

The original contributions presented in the study are included in the article/Supplementary material, further inquiries can be directed to the corresponding author.

## Footnotes

1
https://www.wjx.cn/
